# Quantitative multi-image analysis in metals research

**DOI:** 10.1557/s43579-022-00265-7

**Published:** 2022-10-14

**Authors:** M. J. Demkowicz, M. Liu, I. D. McCue, M. Seita, J. Stuckner, K. Xie

**Affiliations:** 1 Department of Materials Science and Engineering, Texas A&M University, College Station, TX 77843 USA; 2 Physics and Engineering Department, Washington and Lee University, Lexington, VA 24450 USA; 3 Department of Materials Science and Engineering, Northwestern University, Evanston, IL 60208 USA; 4 School of Mechanical and Aerospace Engineering, Nanyang Technological University, Singapore, 639798 Singapore; 5 School of Materials Science and Engineering, Nanyang Technological University, Singapore, 639798 Singapore; 6 Materials and Structures Division, NASA Glenn Research Center, 21000 Brookpark Rd, Cleveland, OH 44135 USA

## Abstract

Quantitative multi-image analysis (QMA) is the systematic extraction of new information and insight through the simultaneous analysis of multiple, related images. We present examples illustrating the potential for QMA to advance materials research in multi-image characterization, automatic feature identification, and discovery of novel processing-structure–property relationships. We conclude by discussing opportunities and challenges for continued advancement of QMA, including instrumentation development, uncertainty quantification, and automatic parsing of literature data.

**Graphical abstract:**

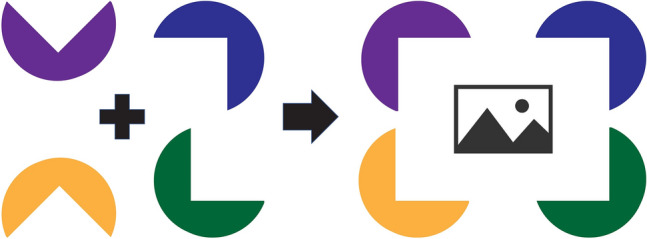

**Supplementary Information:**

The online version contains supplementary material available at 10.1557/s43579-022-00265-7.

## Motivation

Images are among the most information-rich forms of data acquired in materials research. They are often presented individually, as the final output of a single observation, intended for direct visual inspection or quantitative analysis. However, as anyone who has seen a movie or viewed a stereograph can attest, images provide even more insight when grouped, rather than treated in isolation. We define quantitative multi-image analysis (QMA) as the systematic extraction of new information and insight through the simultaneous analysis of multiple, related images. This paper illustrates the potential for QMA to revolutionize materials research by transforming the way we use images. Our examples are drawn from metals research, but employ techniques that are transferrable to other material classes.

**Figure 1:**
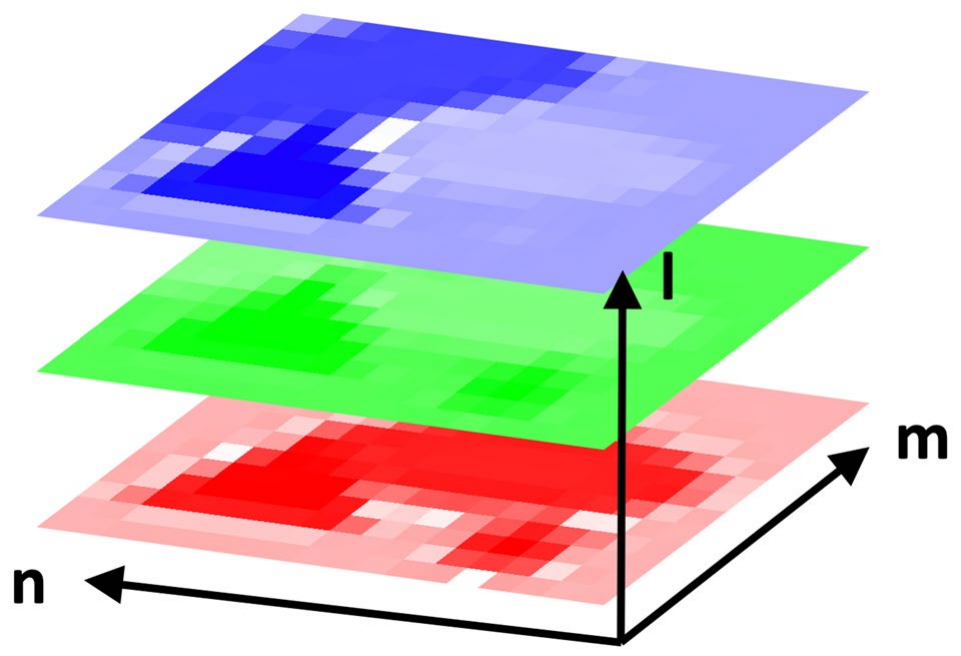
An image is a grid of *m* × *n* locations, each of which has a stack of *l* numerical values associated with it.

An image is fundamentally a *l* × *m* × *n* array of numbers, as illustrated in Fig. 1. The array encodes a *m* × *n* grid of locations and *l* different quantities for each location (for example, *l* = 1 in a grayscale image and *l* = 3 for RGB). One objective of image analysis is to identify features within the *m* × *n* grid of locations, e.g., through edge detection, segmentation, skeletonization, or chord-length distributions.^[1,2]^ Another is to elicit insights from the *l* different data streams at each location, as in the inherently multi-modal images obtained by electron,^[3–8]^ scanning probe,^[9]^ and correlative^[10,11]^ microscopy. QMA goes beyond these traditional forms of analysis by combining information from multiple images to improve statistical inferences, recognize feature evolution, or increase the number of data streams, *l*, at each location.

Automation is essential for QMA: it makes simultaneous analysis of multiple image fast and reproducible,^[12]^ reduces noise and errors,^[13]^ and may be easily accomplished using ready available software, such as ImageJ^[14]^ or MIPAR.^[15]^ Moreover, it opens opportunities to employ computationally intensive techniques such as dimensionality reduction^[16,17]^ and neural networks^[3]^ to extract insights that are otherwise unobtainable. Therefore, our paper highlights automated computational methods for QMA.

## Multi-image characterization of a single sample location

**Figure 2:**
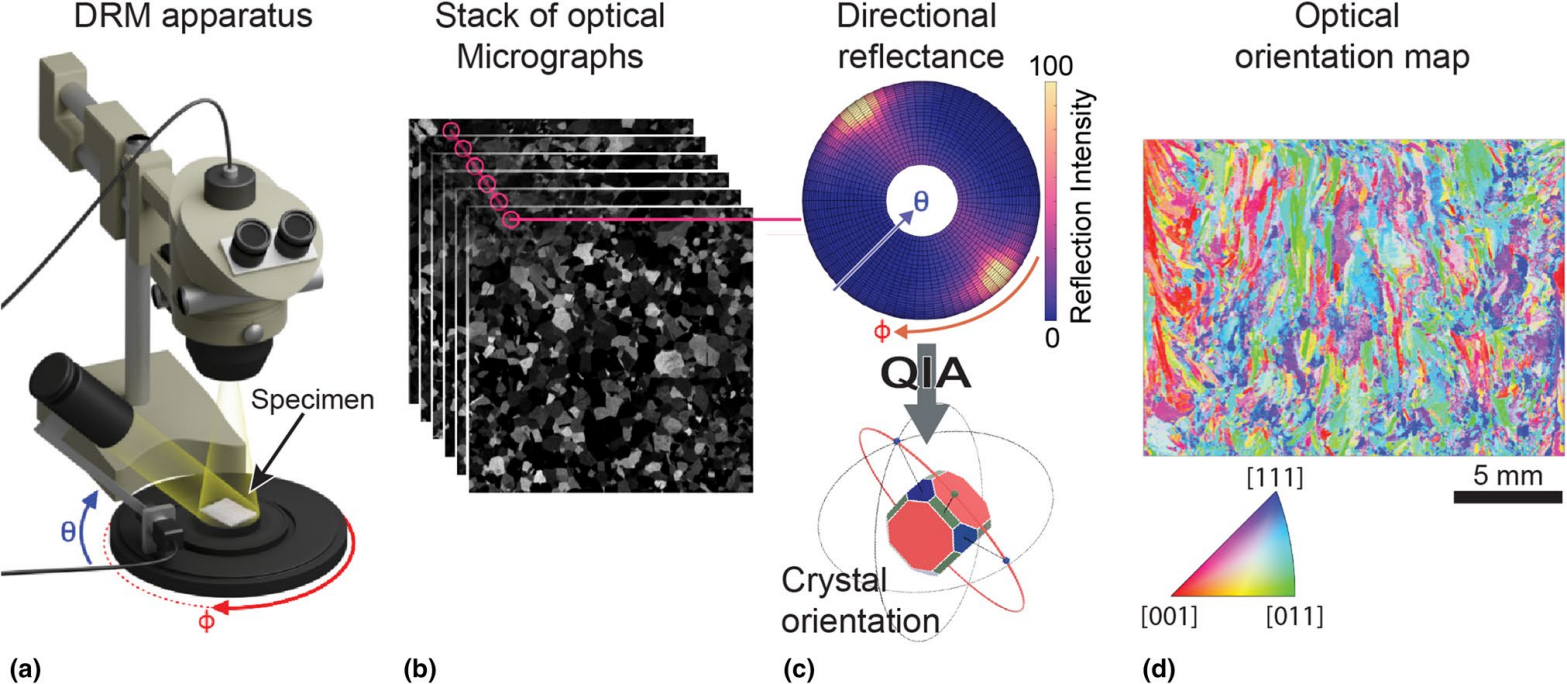
DRM workflow (adapted from Refs. 19–21). (a) A typical DRM apparatus includes a stereo-microscope and motorized stage, which is used to control the illumination direction (parameterized by the elevation, $\it \theta$, and azimuth, $\it \varphi$, angles). (b) Stack of optical micrographs taken for different combinations of $\it \theta$ and $\it \varphi$. (c) Directional reflectance signal from a single pixel in the DRM dataset (b). The signal quantifies how the pixel’s reflected light intensity changes as a function of $\it \theta$ and $\it \varphi$. Analysis of this information enables computing the local crystallographic orientation at the pixel. (d) Example DRM orientation map of an Inconel 718 sample produced using directed energy deposition technology. The map shows crystal orientation along the out-of-plane direction using the standard inverse pole figure color-coding. However, DRM can also measure the full three-dimensional crystal orientation.

*N* images with one data stream taken at a single sample location, but with distinct imaging conditions, may be “stacked” to form a single image with *l* = *N* data streams. Using this approach, directional reflectance microscopy (DRM) obtains microstructure information from optical micrographs that would otherwise only be accessible in electron microscopy.^[18]^ In polycrystals, local surface reflectivity is a function of the crystallographic orientation of individual grains. This phenomenon is the basis of classical metallography. However, inspection of a single micrograph permits only the qualitative conclusion that certain areas possess different crystal orientations and therefore belong to different grains. By contrast, DRM further recognizes that a polycrystal reflects light with varying intensity depending on illumination direction.^[10]^ By stacking multiple micrographs taken at a single location over a range of illumination angles, as shown in Fig. 2, DRM determines the orientation of each grain quantitatively.

Depending on the complexity of the optical signal, different algorithms may be employed to index crystallographic orientation. In some cases, it is sufficient to use computer vision to recognize specific signal patterns that are related to the underlying crystal structure [Fig. 2(c)].^[19]^ In other cases, the measured signals may be compared against a dictionary of simulated ones, modeled through first-principal calculations, in search for the best match.^[22]^ When the interpretation of the optical signal becomes more challenging—for example in multi-phase metal alloys—machine learning (ML) can establish the links between directional reflectance and crystal orientation.^[21]^ Both types of algorithms are able to yield grain orientation maps that are essentially equivalent to those provided by electron backscatter diffraction (EBSD),^[23]^ but over much greater sample areas and without having to place the sample in a vacuum chamber.

DRM has been demonstrated on pure metals,^[20,24]^ semiconductors,^[19]^ metal alloys,^[21]^ and ceramic reinforced polymer composites.^[25]^ However, it holds greatest promise wherever conventional characterization techniques, such as EBSD, struggle to assess large-scale microstructure variability, e.g., in additively manufactured materials.^[26,27]^ Other techniques that integrate multiple images to expand the microstructure information obtainable through optical microscopy include ones that vary light polarization to infer crystallographic textures^[28–30]^ or, in some cases, full crystal orientations.^[31]^ Crystallographic textures may also be inferred from topographic analysis.^[32]^

Another form of QMA correlates single-stream images taken at one location, but with distinct physical conditions imposed on the sample. For example, digital image correlation (DIC) computes local strains by comparing images under different imposed mechanical loads. Originally developed for 2D in-plane analysis with an optical camera,^[33,34]^ DIC has now been applied to analyze images from SEM^[35]^ and AFM,^[36]^ out-of-plane measurement based on binocular stereovision,^[37]^ as well as 3D x-ray computed tomography^[38]^ (digital volumetric correlation). Strain fields obtained through DIC reveal local deformation behavior, such as planar slip and grain boundary sliding.^[39]^ Supplementary Movie 1 illustrates multiple slip band formation in a Ni-base alloy during an in situ SEM tensile test.

Quantitative processing of DIC data provides new ways to understand material behavior at the microstructural level. For example, the Heaviside-DIC algorithm identifies displacement discontinuities due to localized slip.^[40]^ Moreover, DIC data may be registered with the underlying microstructure (as characterized by EBSD) and analyzed by ML to identify active twinning systems,^[41]^ predict strain at grain boundaries with different orientations relative to a loading axis,^[42]^ as well as distortions near defects.^[43]^

## Feature extraction through interpolation of image databases

Identification of features in an image may be posed as a multi-dimensional, nonlinear interpolation problem on a database of representative images where the features of interest have been identified in advance. For example, the *DefectSegNet* code^[44]^ automatically identifies crystal defects, such as dislocations^[45]^ or voids,^[46]^ in transmission electron microscopy (TEM) images. It requires a database of TEM images where the defects of interest have been previously identified, either via inspection by an expert or through automated image analysis (e.g., using the Sauvola method for dislocations^[47]^ or subtraction of underfocus and overfocus images for voids^[48]^). These images are taken to be a representative sampling of a hypothetical set of all possible TEM micrographs containing the defects of interest.

**Figure 3:**
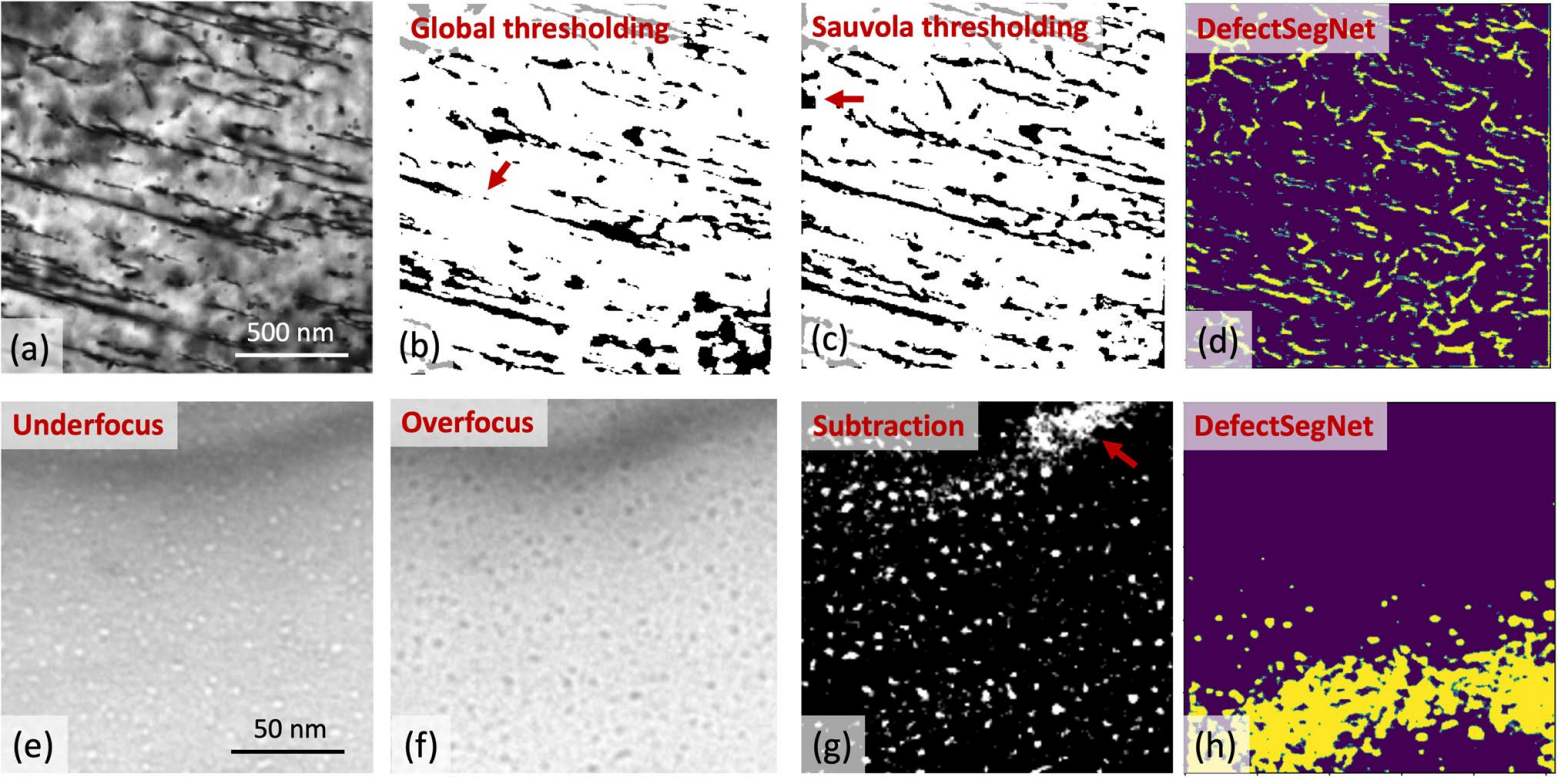
(a) TEM micrograph showing dislocations in Mg. Dislocation lines were identifies using (b) global thresholding, (c) the Sauvola method (a local thresholding method), and (d) *DefectSegNet*. TEM micrographs showing helium bubbles in Cu in (e) underfocus and (f) overfocus imaging conditions. Bubbles were detected using (g) the subtraction method and (h) *DefectSegNet*. Red arrows indicate locations where diffraction contrast artifacts make it difficult to currently identify dislocations or bubbles.

For any given new TEM image, *DefectSegNet* generates a semantic segmentation image identifying defect pixels through interpolation of the database using a convolutional neural network (CNN), which is a type of machine learning algorithm for analyzing images. This method yields high accuracy, capable of detecting ~ 92% dislocations and ~ 99% voids correctly in TEM images where defects are clearly visible on a clean background.^[44]^ Performance is poorer on micrographs that contain diffraction contrast artifacts (e.g., lattice distortion or bending contours). Figure 3 applies *DefectSegNet* to two example TEM images and compares it to conventional algorithms. The latter currently outperform *DefectSegNet*, but larger training sets that include images with diffraction contrast artifacts may mitigate this shortcoming of *DefectSegNet*.

## Mining processing–structure–property relationships

**Figure 4:**
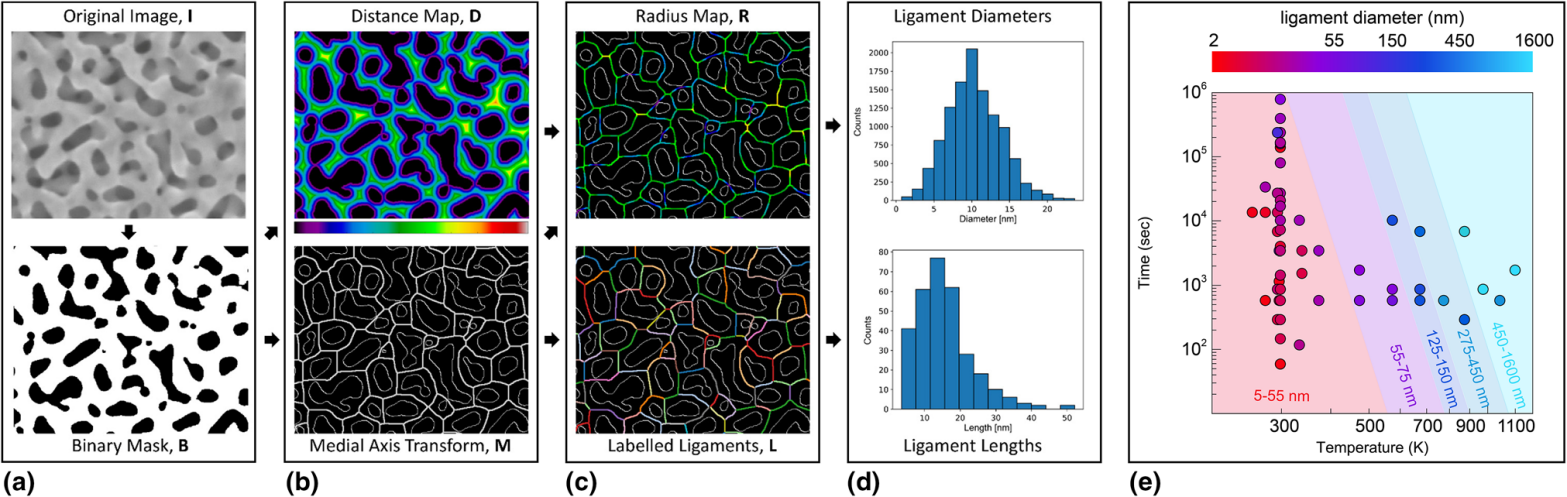
(a) Original (top) and thresholded (bottom) SEM micrograph of nanoporous gold (NPG). (a)–(d) The AQUAMI code performs quantitative image analysis through noise removal, pixel-by-pixel segmentation, and measurement (here, ligament diameter and length). (e) Time–temperature processing map of NPG constructed using image data and metadata mined from the open literature. The boundaries between colored regions represent approximate isocontours of ligament diameter.

Image databases augmented with metadata that summarizes associated processing conditions or material properties may be analyzed to refine known processing–structure–property (PSP) relationships or discover new ones. Images published in the open literature constitute such a database, albeit not an easily searchable one. For example, after inspecting approximately 1000 manuscripts, McCue et al*.* identified 128 published studies reporting processing histories as well as high quality images for nanoporous gold (NPG): a well-studied material fabricated via selective dissolution of Ag from a AgAu alloy^[49]^ [see Fig. 4(a)]. Using the AQUAMI software,^[50]^ they characterized NPG microstructure consistently across all images and discovered quantitative relationships between NPG processing parameters (time and temperature) and ligament diameters, as shown in Fig. 4.

**Figure 5:**
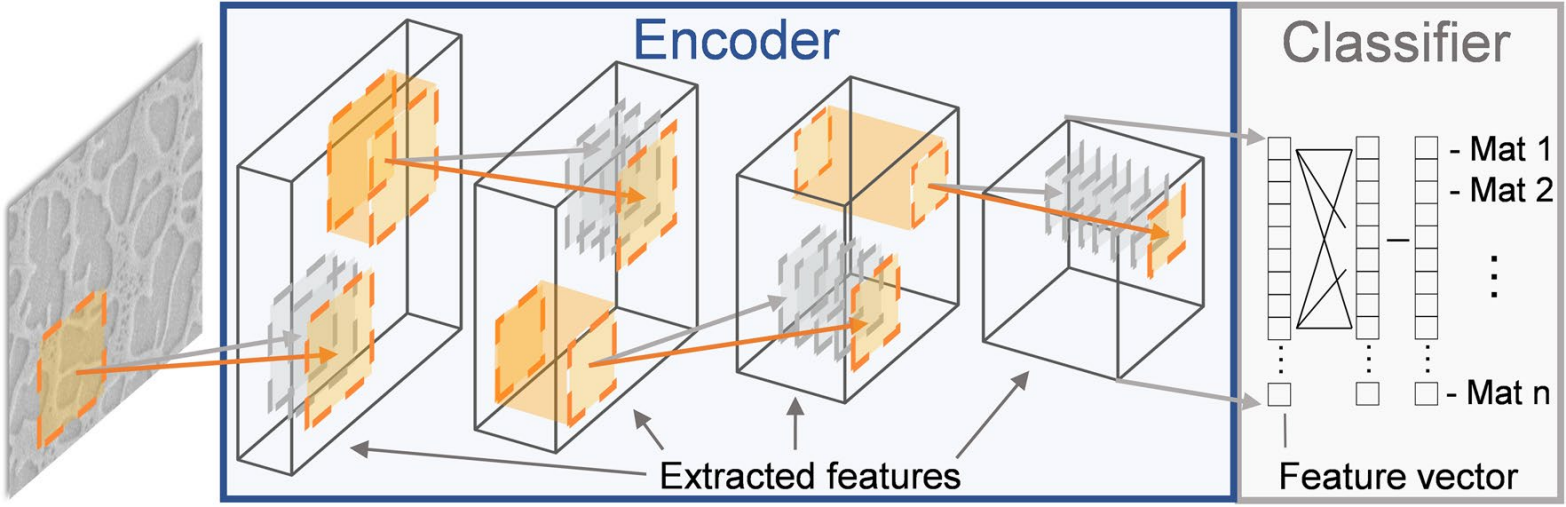
A typical convolutional neural network (CNN) consists of an encoder (outlined in blue) which extracts increasingly high-level features from an image in successive layers. The model also has a task specific head (in this case a classifier outlined in grey), which uses the feature vector generated from the encoder to perform image analysis tasks such as classification, segmentation, or property prediction.

A common objective of data mining studies, such as the example given above, is to distill information into trends that can be easily understood by humans.^[51–54]^ CNNs are also capable of integrating microscopy images and processing information to make property predictions.^[55]^ A CNN, as illustrated in Fig. 5, extracts quantitative features from the micrograph and transforms the high-dimensional image into a useful lower dimensional representation. CNNs contain an encoder with many layers which successively extract more complex information from the image starting with features such as edges and textures in the initial layers to whole objects in the later layers. Each layer has many image filters which detect a single feature in the image. Similar to the *DefectSegNet* code^[44]^ described in section “Feature extraction through interpolation of image databases”, such CNNs must be trained on a suitable database of images and associated metadata to determine appropriate feature extraction filters for the desired task. Pre-training encoders on a massive dataset of microscopy images improves performance on segmentation tasks when little training data is available, and the models are more robust to changes in imaging and sample conditions.^[56]^ The quantitative information extracted by the CNN can be used to establish PSP relationships.^[57–61]^

## Discussion

The foregoing examples illustrate the potential for QMA to advance materials research. They also shed light on challenges and opportunities for continued development of QMA methods. The first of these concerns physical tools. Today, multi-image characterization at individual sample locations (Sect. 2) is often carried out using custom-built tools. Incorporation of capabilities for varying illumination conditions—including angles of incidence, polarization, or wavelength—into commercial optical microscopes has the potential to advance QMA methods and make them more accessible to a broader community of researchers. In the case of DIC, while commercial tools are available for optical characterization, SEM-based DIC requires in-house integration of loading, imaging, and analysis. Moreover, there are currently no tools for out-of-plane displacement measurement in SEM-DIC, limiting its utility in characterizing surface topography changes, e.g., pile-up regions near micro- or nano-indents.^[62]^ This shortcoming may be addressed by acquiring stereo SEM images using a tilting stage or by integrating SEM and AFM data.

Conclusions drawn from QMA require quantification of uncertainty. This topic has received considerable attention within the DIC community, due to inherent tradeoffs between smoothing of image noise and the resolution of the calculated strain map.^[6,62,63]^ Uncertainty quantification challenges also arise in automated feature detection. For example, orientation and strain mapping in precession electron diffraction (PED)^[64,65]^ and 4D-STEM^[66]^ datasets (e.g., to detect geometrically necessary dislocations^[67]^) require precisely locating the centers of diffracted spots with non-uniform intensity.^[68]^ This task calls for assessing the impact of approximations made during analysis on the resulting conclusions. This challenge also arises in TEM characterization of beam-sensitive materials, where very low electron doses are required to minimize electron-beam-induced damage. This constraint leads to noisy datasets that must be filtered using advanced statistical algorithms.^[69,70]^

Some of the QMA examples we gave require image databases. The materials informatics community is rapidly automating methods to identify features and extract PSP relationships from such databases.^[53,71,72]^ This work mirrors the imaging revolution that previously occurred in medicine, where content-based image retrieval now aids in patient diagnosis and care.^[52,73]^ However, labeled and parsed materials databases are limited in size and scope, focusing on specific subsets of metadata.^[74–77]^ The most comprehensive set of data is still located in published manuscripts. There is a growing effort to parse, archive, and access microstructural data from the literature,^[49]^ though automation of this task is only nascent.^[78]^ Construction of image databases with web-scale quantities of data will enable discovery of new PSP relationships. This undertaking will undoubtedly benefit from continued development of CNNs and vision transformers.^[79–82]^

## Supplementary Information

Below is the link to the electronic supplementary material.

Supplementary file1 (MP4 1691 kb).
